# Supplementary material for: From sequence to enzyme mechanism using multi-label machine learning
Source: BMC Bioinformatics. 2014 May 19;15:150. doi: 10.1186/1471-2105-15-150 (PMC4229970; doi:10.1186/1471-2105-15-150)
Supplement: Additional file 2 — Java code of ml2db. Additional file ml2db_code.tar.gz contains the Java source code to run the multi-label machine learning experiments and save the results to database. The code’s Javadoc is included. [file 1471-2105-15-150-S2.zip › additional file 2/ml2db/utils/doc/uk/ac/ed/inf/utils/maputils/IndexedOneToManyMap.html]

IndexedOneToManyMap


---


|  |  |  |
| --- | --- | --- |
| **Overview** \| **Package** \| **Class** \| **Use** \| **Tree** \| **Deprecated** \| **Index** \| **Help** | |  |
| PREV CLASS   **NEXT CLASS** | **FRAMES**    **NO FRAMES**     **All Classes** |  |
| SUMMARY: NESTED \| FIELD \| CONSTR \| METHOD | DETAIL: FIELD \| CONSTR \| METHOD |  |


---


## uk.ac.ed.inf.utils.maputils Class IndexedOneToManyMap<T,U>

```
java.lang.Object
  java.util.AbstractMap<K,V>
      java.util.TreeMap<T,java.util.Set<U>>
          uk.ac.ed.inf.utils.maputils.OneToManyMap<T,U>
              uk.ac.ed.inf.utils.maputils.IndexedOneToManyMap<T,U>
```

**All Implemented Interfaces:**: java.io.Serializable, java.lang.Cloneable, java.util.Map<T,java.util.Set<U>>, java.util.NavigableMap<T,java.util.Set<U>>, java.util.SortedMap<T,java.util.Set<U>>

---

``` public class IndexedOneToManyMap<T,U> extends OneToManyMap<T,U> ```

A one-to-many map that uses an internal integer index to save memory. Useful,
for example, for maps having few, long-string repeated values. Do not use if
you need to add or remove individual objects. This map cannot take an empty
or null string as key or value.

**Version:**
:   28 Mar 2011

**Author:**
:   Luna De Ferrari luna.deferrari-at-ed.ac.uk

**See Also:**: Serialized Form

---

**Nested Class Summary**

**Nested classes/interfaces inherited from class java.util.AbstractMap**

`java.util.AbstractMap.SimpleEntry<K,V>, java.util.AbstractMap.SimpleImmutableEntry<K,V>`


**Constructor Summary**

`IndexedOneToManyMap(java.util.Vector<T> keys,
java.util.Vector<U> values)`
  
          Initialise with a list of key-value pairs


| **Method Summary** | |
| --- | --- |
| `boolean` | `containsKeyValue(T key, U value)`             Checks if the map contains a specific key-value pair |
| `boolean` | `containsValue(U value)`             Checks if the map contains a value |
| `java.util.Set<U>` | `getAKeyValues(T key)`             Get the set of unique values for this key. |
| `java.util.Set<U>` | `getNonEmptyValues()`             Get the set of unique, non-empty-string values for this map. |
| `java.util.Set<U>` | `getValues()`             Get the set of unique values for this map. |
| `void` | `removeValue(T key, U value)`             Remove a value from the key's list of values |

**Methods inherited from class uk.ac.ed.inf.utils.maputils.OneToManyMap**

`addAll, addAll, addKeyAndValue, clear, containsKey, containsValue, deleteKeyValue, generateAKeyValues, getKeysVectorValuesVector, getKeysWithValue, getKeyValuesPairsSize, getNonEmptyKeys, getNonEmptyKeyValuesPairsSize, getRandomValue, invertKeysAndValues, isEmpty, mapIsNullOrEmpty, removeKey, removeValue, removeValues, toCSV, toString`

**Methods inherited from class java.util.TreeMap**

`ceilingEntry, ceilingKey, clone, comparator, descendingKeySet, descendingMap, entrySet, firstEntry, firstKey, floorEntry, floorKey, get, headMap, headMap, higherEntry, higherKey, keySet, lastEntry, lastKey, lowerEntry, lowerKey, navigableKeySet, pollFirstEntry, pollLastEntry, put, putAll, remove, size, subMap, subMap, tailMap, tailMap, values`

**Methods inherited from class java.util.AbstractMap**

`equals, hashCode`

**Methods inherited from class java.lang.Object**

`getClass, notify, notifyAll, wait, wait, wait`

**Methods inherited from interface java.util.Map**

`equals, hashCode`

**Constructor Detail**

### IndexedOneToManyMap

```
public IndexedOneToManyMap(java.util.Vector<T> keys,
                           java.util.Vector<U> values)
```

:   Initialise with a list of key-value pairs

    **Parameters:**: `keys` - the list of keys: `values` - the list of values (the value for each key in the keys list)


**Method Detail**

### containsKeyValue

```
public boolean containsKeyValue(T key,
                                U value)
```

:   Checks if the map contains a specific key-value pair

    :   **Overrides:**: `containsKeyValue` in class `OneToManyMap<T,U>`
    :   **Parameters:**: `key` -: `value` - **Returns:**: true if the key-value exists, false otherwise (false if the key or value are null)

---


### containsValue

```
public boolean containsValue(U value)
```

:   Checks if the map contains a value

    :   **Parameters:**: `value` - a value **Returns:**: true if the value exists in the map, false otherwise

---


### getAKeyValues

```
public java.util.Set<U> getAKeyValues(T key)
```

:   Get the set of unique values for this key. No values can be null. At most
    one of the values can be an empty string.

    :   **Overrides:**: `getAKeyValues` in class `OneToManyMap<T,U>`
    :   **Returns:**: the set of unique values for this key

---


### getNonEmptyValues

```
public java.util.Set<U> getNonEmptyValues()
```

:   Get the set of unique, non-empty-string values for this map.

    :   **Overrides:**: `getNonEmptyValues` in class `OneToManyMap<T,U>`
    :   **Returns:**: the set of unique, non-empty-string values for this map

---


### getValues

```
public java.util.Set<U> getValues()
```

:   Get the set of unique values for this map. At most one value can be null.
    At most one value can be an empty string.

    :   **Overrides:**: `getValues` in class `OneToManyMap<T,U>`
    :   **Returns:**: the set of unique values for this map

---


### removeValue

```
public void removeValue(T key,
                        U value)
```

:   Remove a value from the key's list of values

    :   **Overrides:**: `removeValue` in class `OneToManyMap<T,U>`
    :   **Parameters:**: `key` -: `value` -


---


|  |  |  |
| --- | --- | --- |
| **Overview** \| **Package** \| **Class** \| **Use** \| **Tree** \| **Deprecated** \| **Index** \| **Help** | |  |
| PREV CLASS   **NEXT CLASS** | **FRAMES**    **NO FRAMES**     **All Classes** |  |
| SUMMARY: NESTED \| FIELD \| CONSTR \| METHOD | DETAIL: FIELD \| CONSTR \| METHOD |  |


---
